# Supplementary material for: Shared decision-making between patients and healthcare providers at rural health facilities in Eastern Uganda: an exploratory qualitative study
Source: BMC Med Ethics. 2025 Jan 27;26:13. doi: 10.1186/s12910-025-01172-x (PMC11773793; doi:10.1186/s12910-025-01172-x)
Supplement: Supplementary file 1 — Supplementary Material 1. [file 12910_2025_1172_MOESM1_ESM.pdf]

**IN- DEPTH INTERVIEW GUIDE FOR HEALTHCARE PROVIDERS.**

**STUDY TITLE:** Experiences and ethical issues during shared decision-making in healthcare: A case of Budumba Health Centre III and Butaleja HC III.

**Participant study ID:_ _ _ _ _ _ _ _ _ _ _**

a) Age …………………….

b) Gender ………………

c) Position ………………………...

1. Briefly describe your position/ responsibility.

2. How long have you held this position?

3. Can you please describe your understanding of the term shared decision-making? *Probe: Shared decision-making is said to be associated with better treatment outcomes, what is your view on this?*

4. Please share with me your experience having shared decision with patients about their healthcare? *Probe: Are there instance where a patient refused to engage in decision-making? If so, what do you do, then?*

5. What are some of the challenges you encountered as you engage the patients to take part in shared decision-making? *Probe for: Patient, facility and healthcare provider factors.*

6. What are some of the ethical issues that emerge as you involve patients in decision-making?

7. In your view, what strategies could help to improve shared decision-making processes in your setting.

8. Any comments, suggestion or observations you would like to make about shared decision making in rural health care?

***Thank you for your time***
