# Supplementary material for: Shared decision-making between patients and healthcare providers at rural health facilities in Eastern Uganda: an exploratory qualitative study
Source: BMC Med Ethics. 2025 Jan 27;26:13. doi: 10.1186/s12910-025-01172-x (PMC11773793; doi:10.1186/s12910-025-01172-x)
Supplement: Supplementary file 2 — Supplementary Material 2. [file 12910_2025_1172_MOESM2_ESM.pdf]

# IN- DEPTH INTERVIEW GUIDE FOR PATIENTS.

**STUDY TITLE:** Experiences and ethical issues during shared decision-making in healthcare: A case of Budumba Health Centre III and Butaleja HC III.

**Participant study ID:_ _ _ _ _ _ _ _ _ _ _**

a) Age …………………….

b) Gender ………………

c) Position ………………………...

d) Occupation……………………

1. As a patient, what do you understand by shared decision-making in relation to your healthcare?

2. How do you feel about being engaged in shared decision making, and do you think healthcare providers should always engage you? *Why do you think patients should/should not be engaged be in health care decisions?*

3. Kindly share with me your experience with healthcare providers during shared decision-making? *Probe: Are there cases in that you feel the healthcare provider did not engage you in decision-making or you were not involved in decision-making about your health?*

4. What barriers do you encounter when sharing decision with your healthcare provider? *Probe for: Patient, facility, and social factors*

5. In your view, what do you think should you be done to improve patient involvement in decision regarding their health? *Probe for possible: Patient, healthcare provider, facility strategies to improve shared decision making in healthcare*

6. Do you have any other comments or observations you would like to make regarding healthcare providers involving patients in shared decision-making?

***Thank you for your time***
